# Oral cadmium exposure to environmental doses induces visceral adiposopathy in Wistar rats

**DOI:** 10.1007/s00204-025-04228-4

**Published:** 2025-11-07

**Authors:** Victor Enrique Sarmiento-Ortega, Diana Moroni-González, Alfonso Diaz, Eduardo Brambila, Samuel Treviño

**Affiliations:** 1 Laboratory of Metabolomic and Chronic Degenerative Diseases, Physiology Institute, Meritorious Autonomous University of Puebla, Prol. de la 14 Sur 6301, Ciudad Universitaria, C.P. 72560 Puebla, Mexico; 2 Laboratory of Chemical-Clinical Investigations, Department of Clinical Chemistry, Chemistry Department, Meritorious Autonomous University of Puebla, 14 Sur. FCQ1, Ciudad Universitaria, C.P. 72560 Puebla, Mexico; 3 Laboratory of Neurochemistry and Behavior, Physiology Institute, Meritorious Autonomous University of Puebla, Prol. de la 14 Sur 6301, Ciudad Universitaria, C.P. 72560 Puebla, Mexico; 4 Faculty of Nursing, Meritorious Autonomous University of Puebla, Av. 25 Pte. 1304, C.P. 72410 Puebla, Mexico

**Keywords:** Cadmium toxicity, Adiposopathy, Inflammation, Macrophage polarization, Fibrosis

## Abstract

Chronic cadmium exposure, even in environmental doses, has been linked to multiple metabolic disturbances, including white adipose tissue (WAT) dysfunction. WAT dysfunction is defined as a loss of endocrine, immunologic, and metabolic homeostasis, characterized by a low-grade, progressive, and non-resolving inflammation development, namely adiposopathy. This study evaluated the immunometabolic effects of Cd exposure in drinking water on WAT of male Wistar rats, using concentrations of 15 and 32 ppm (environmental doses) over periods of up to 5 months. Inflammatory markers in serum and tissue were analyzed, along with macrophage phenotype, NF-κB pathway activation, leptin and adiponectin expression, correlations with the adiponectin/leptin (A/L) index, and the development of fibrosis. The results showed a progressive increase in proinflammatory cytokines (IL-6, TNF-α, IL-1β), sustained NF-κB activation, and a shift from anti-inflammatory (CD206⁺) to proinflammatory (CD16⁺) macrophages. These changes were accompanied by dysregulation of the adiponectin/leptin axis and a decrease in the A/L ratio, with dynamic correlations to immune markers. Fibrosis was detected in late stages. In conclusion, our results demonstrated for the first time that Cd exposure in environmental doses induces adiposopathy; thereby, findings indicate that Cd can progressively disrupt the immunometabolic homeostasis of adipose tissue, promoting an inflammatory and profibrotic environment with potential implications for the development of metabolic diseases.

**Supplementary Information:**

The online version contains supplementary material available at 10.1007/s00204-025-04228-4.

## Introduction

Environmental pollution by heavy metals represents a growing threat to global public health. Industrial, agricultural, and mining activities have significantly increased this type of pollution, posing a significant health risk (Dagdag et al. 2023). Cadmium (Cd) is recognized as one of the most toxic contaminants, primarily released through industrial activities, vehicular emissions, the use of phosphate fertilizers, and tobacco smoke (Yuan et al. 2019; Kubier et al. 2019). The Agency for Toxic Substances and Disease Registry (ATSDR) has defined the environmental Cd concentrations as the minimal risk level of daily exposure to metal that has no appreciable risk of adverse effects, such as cancer development. These are the no observable adverse effect level (NOAEL; < 1 μg of Cd/kg/day) and lowest observable adverse effect level (LOAEL; 1 μg to 2 mg of Cd/kg/day). Therefore, international organizations recommend in their guidelines a maximum Cd intake limit in drinking water (0.5 μg/kg/day) and in food (1 μg/kg/day). Due to Cd being progressively accumulated in tissues such as the kidney, liver, lung, bone, and adipose tissue, it has high bioavailability and a long half-life in the body, thereby exerting toxic effects (Satarug et al. 2010, 2023; Thévenod and Lee 2013; Sarmiento-Ortega et al. 2025a).

Chronic Cd exposure has been associated with a broad spectrum of diseases, including kidney and liver dysfunction, reproductive disorders, cardiovascular disease, cancer, and metabolic disturbances, many of them mediated by oxidative stress, inflammation, and persistent cellular damage (Edwards and Prozialeck 2009; Satarug et al. 2010; Luevano and Damodaran 2014; Edwards and Ackerman 2016; Sarmiento-Ortega et al. 2017, 2022, 2023; Das and Al-Naemi 2019; Chung and Chang 2025). However, recently, it has been questioned whether Cd exposure is related to obesity and adipose tissue dysfunction (Tinkov et al. 2017; Attia et al. 2022b, a; Sarmiento-Ortega et al. 2022, 2025b; Satarug 2023; Zhu et al. 2024).

White adipose tissue (WAT), traditionally considered an energy reservoir that provides insulation for the body, plays roles in thermoregulation and mechanical organ protection. Still, today it is recognized as an endocrine and immunologically active organ too. Its participation in energy and metabolic homeostasis depends on a delicate interaction between adipocytes, immune cells, and extracellular matrix (Yang Loureiro et al. 2022; Huang et al. 2024). WAT is predominantly located in subcutaneous and visceral depots, with smaller amounts found in bone marrow and muscle tissue. Visceral WAT comprises 10–20% of total body fat in men and 5–10% in women; however, it is also more prone to inflammation, contributing to a higher risk of chronic diseases. In rodents, the epididymis is the most studied visceral WAT because its dysfunction is a hallmark of metabolic and chronic-degenerative diseases (Lopez-Yus et al. 2024; Blüher 2024).

The hallmarks of visceral WAT dysfunction can be summarized as adipocyte hypertrophy with adiposopathy: hyperleptinemia, hypoadiponectinemia, selective adipocyte insulin resistance, immune cell infiltration of pro-inflammatory macrophage and lymphocyte that induce fibrosis, and conditions that contribute to ectopic fat deposition (in the liver, pancreas, and other organs), diabetes development, and atherogenic conditions (Reyes-Farias et al. 2021; Sarmiento-Ortega et al. 2025b). M1 pro-inflammatory macrophages in dysfunctional visceral WAT under chronic overproduce cytokines such as interleukin 6 (IL-6), tumor necrosis factor α (TNF-α), and interleukin 1-β (IL-1β), altering the secretion of adipokines such as leptin and adiponectin (Kawai et al. 2021; Kolb 2022; Tilg et al. 2025). In addition, chronic inflammation favors fibrosis, a condition that stiffens the architecture of adipose tissue, limits its functional expansion, and impairs its metabolic response capacity (Datta et al. 2018; Gliniak et al. 2023).

In this context, research on the effects of Cd on adipose tissue is still incipient, despite its potential to induce metabolic dysfunction. The work aims to deepen this relationship by evaluating the immunological, structural, and functional epididymal WAT alterations of male Wistar rats induced by chronic exposure to environmental doses of Cd, across subacute, subchronic, and chronic periods, with a focus on changes in inflammatory responses, macrophage polarization, fibrosis, and the dysregulation of leptin/adiponectin production.

## Materials and methods

### Animals and treatment

Ninety male Wistar rats (initial weight: 70–80 g) were procured from the “Claude Bernard” animal facility at the Universidad Autónoma de Puebla (Puebla, Mexico). Animals were housed in a temperature-controlled environment (22 °C), under a 12-h light/dark cycle, with unrestricted access to standard rodent chow and water. Animals were maintained on a balanced diet (5001, LabDiet; St. Louis, MO, USA) until reaching a body weight of approximately 200 g. Then, rats were randomly assigned to control (n = 30) and experimental groups (n = 60). The control group received Cd-free drinking water ad libitum, whereas the experimental groups were exposed to cadmium chloride (CdCl₂) via drinking water at concentrations of 15 ppm and 32 ppm. Control and experimental groups were evaluated at six different time points: 15 days, 1, 2, 3, 4, and 5 months (n = 5 per group). At the end of each experimental period, animals were fasted for 4–5 h before sample collection. Blood was obtained via cardiac puncture under deep anesthesia induced with a xylazine/ketamine combination (20/137 mg/kg). Serum was obtained by centrifugation and stored at − 70 °C until analysis. Epididymal adipose tissue was excised, rinsed with cold saline to remove residual blood, and either frozen at − 70 °C or fixed in 10% neutral buffered formalin for histological processing. All experimental procedures conformed to the ethical guidelines established by the Mexican Official Standard NOM-062-ZOO-1999 and were approved by the Institutional Animal Care and Use Committee (CICUAL: UALVIEP 22/2). The study was conducted in full accordance with national and international standards for laboratory animal welfare, and all efforts were made to reduce animal use and minimize distress or discomfort throughout the protocol.

### Biochemical assays in serum

Serum levels of TNF-α, IL-6, IL-1β, interleukin 10 (IL-10, leptin, and adiponectin were determined using ELISA commercial kits (Merck Millipore; Toluca, Mexico) and quantified in a Stat fax 2600 plate reader at 415 nm (WinerLab Group, Buenos Aires, Argentina). The adiponectin/leptin index (A/L index) was calculated by dividing the adiponectin concentration by the leptin concentration (Frühbeck et al. 2018).

### Adipose tissue techniques

#### CD206^+^ and CD16^+^ cells immunofluorescence staining

According to standard procedures, paraffin-embedded adipose tissue Sects. (9 µm thick) were deparaffinized and rehydrated. For immunofluorescence staining, tissue sections were subjected to antigen retrieval procedures. Subsequently, sections were incubated with CD206 and CD16 primary antibodies (Santa Cruz Biotechnology Inc., CA, USA), followed by a rhodamine-conjugated secondary antibody and a fluorescein isothiocyanate (FITC)- conjugated secondary antibody (Jackson ImmunoResearch Laboratories). Confocal microscopy was performed using a Nikon A1 confocal microscope (Tokyo, Japan); the objective Nikon Plan Apo 20X DIC M N2 (NA 0.75) was used to capture images at 20 × magnification. ImageJ software (NIH) was used for contrast, brightness, and pseudocolor adjustments. Results were expressed as a ratio of positive cells among adipocytes per field.

#### Picrosirius red staining

Picrosirius red staining was performed to evaluate tissue fibrosis. Paraffin-embedded adipose tissue Sects. (9 µm thick) were deparaffinized and rehydrated, incubated in Picrosirius red solution for one hour, and then washed with acidic water. Finally, they were assembled with resinous media. Photomicrographs were captured at 20X magnification using a polarized light microscope (Leica Microsystems GmbH, Wetzlar, Germany). ImageJ software (National Institutes of Health, USA) was used to quantify total fibrosis, which was expressed as the ratio of the area of fibrous tissue stained with picrosirius to the total area of the field (Coelho et al. 2018). The percentage of birefringent tissue was analyzed as the ratio of the area of birefringent tissue to the total area of the field using ImageJ software (National Institutes of Health, USA).

#### Leptin and adiponectin immunofluorescence staining

To perform immunofluorescence staining, tissue sections underwent antigen retrieval procedures. Afterward, the sections were incubated with a primary antibody targeting Leptin (Abcam, Cambridge, MA) and Adiponectin (Cell Signaling Technology, Massachusetts, USA.), followed by treatment with a secondary antibody conjugated to rhodamine (Jackson ImmunoResearch Laboratories) and FITC conjugated secondary antibody, respectively (Jackson ImmunoResearch Laboratories). Fluorescence microscopy equipped with an integrated camera (Leica Microsystems GmbH, Wetzlar, Germany) was utilized to acquire images at a magnification of 20X. For semi-quantitative analysis, ImageJ software (National Institutes of Health, USA) was used, with the results expressed in an arbitrary value.

#### Immunoblotting of NF-κB

Frozen epididymal adipose tissue was homogenized in ice-cold RIPA buffer supplemented with a protease inhibitor cocktail. After centrifugation at 3000 rpm for 10 min at 4 °C, the supernatant containing cytoplasmic proteins was collected. The remaining pellet was processed for nuclear protein extraction using a commercial buffer under constant agitation, followed by centrifugation at 15,000 rpm for 10 min at 4 °C to isolate the nuclear fraction. Protein lysates were mixed with Laemmli buffer and resolved by SDS-PAGE. Electrophoretic transfer to PVDF membranes was performed using Mini Trans-Blot systems (Bio-Rad). Membranes were blocked and incubated overnight at 4 °C with a primary antibody against nuclear factor-κB (NF-κB) (Santa Cruz, CA, USA), followed by incubation with HRP-conjugated secondary antibodies. Signal detection was performed via chemiluminescence (Immobilon Western HRP substrate, Millipore), and bands were visualized using a Nine Alliance Q9 mini photodocumenter. Densitometric analysis was conducted with Image Lab software (Bio-Rad), and band intensities were normalized to Lamin A as a loading control. Relative expression was reported as fold change using the control group (assigned a value of 1) for semi-quantitative comparison.

#### Pro-inflammatory and anti-inflammatory cytokines

Adipose tissue was homogenized in PBS with the addition of protease inhibitors, followed by centrifugation at 2500 × g for 30 min at 4 °C using a 17 TR microcentrifuge (Next Advance, Averill Park, NY). The concentrations of IL-1β, IL-6, TNF-α, IL-10, interleukin 1 receptor antagonist (IL-1Ra), and transforming growth factor beta (TGF-β) in the resulting supernatant were determined using ELISA kits (Merck Millipore; Toluca, Mexico) according to the manufacturer’s instructions. Cytokine levels were normalized to the total protein content and expressed as pg/mg of protein.

### Heatmap

The adiponectin/leptin index was calculated based on the individual concentrations of these adipokines. To assess the relationship between the adiponectin/leptin index and inflammatory biomarkers at different time points throughout the experiment (15 days, 1 month, 2 months, 3 months, 4 months, and 5 months), Pearson’s correlation coefficient was determined using IBM SPSS Statistics. Correlation values were visualized in a correlation map using a bubble plot, where color represents the direction of the correlation (red for positive correlations and blue for negative correlations), and bubble size reflects the strength of the correlation.

### Statistical analysis

The results are presented as the mean ± standard error of the mean (SEM). Data normality was assessed using the Shapiro–Wilk test. Statistical differences between groups for normally distributed data were determined using a one-way analysis of variance (ANOVA). To control for Type I error, post hoc analysis was performed using Bonferroni’s correction for pairwise comparisons against the control group. For data that did not follow a normal distribution (semi-quantitative data), the Kruskal–Wallis test was employed. These analyses were conducted using GraphPad Prism 8 (GraphPad Software Inc., USA). A Pearson’s correlation analysis was conducted to evaluate the linear relationship between specific variables using IBM SPSS Statistics, version 28.0 (IBM Corp., Armonk, NY, USA). All statistical tests were two-tailed, and a significance level of p < 0.05 was considered statistically significant.

## Results

### Impact of cadmium exposure on interleukin and serum hormones

Serum levels of TNF-α, IL-6, IL-1β, IL-10, adiponectin, and leptin were quantified in rats exposed to Cd at 15 ppm or 32 ppm at different time-cohorts (Table 1). At 15 days, only IL-10 increased in both exposure groups: 62.5% (15 ppm) and 72.8% (32 ppm). In the groups exposed for 1 month, the 15 ppm group increased IL-6 (17.3%) and IL-10 (84.9%), while the 32 ppm group increased IL-6 (28.9%), IL-1β (28.5%), and IL-10 (84.9%). By the second month, both groups presented progressive elevations in pro- and anti-inflammatory cytokines, dose-dependent. However, in the 32 ppm group, leptin was also augmented (14.6%). At 3 months, both Cd-exposed groups’ cytokines and leptin levels remained elevated, but adiponectin concentration diminished by 24.4% (15 ppm) and 30.1% (32 ppm). The severe inflammation induced by Cd was observed to persist during the fourth and fifth months of analysis, emphasizing the decline in adiponectin, which is suggestive of adipose dysfunction. Thus, we calculated the A/L index, which significantly diminished from 2 months (15 ppm: 27.3%; 32 ppm: 30.2%), 3 months (15 ppm: 30.0%; 32 ppm: − 36.7%), 4 months (15 ppm: 34.5%; 32 ppm: 44.8%), and 5 months (15 ppm: 36.6%; 32 ppm: 50.7%).

**Table 1:** Effect of cadmium exposure on interleukin and serum hormones

		TNF-α (pg/mL)	IL-6 (pg/mL)	IL-1β (pg/mL)	IL-10 (pg/mL)	Adiponectin (µg/mL)	Leptin (ng/mL)	A/L Index
15 days	Control	14.7 ± 0.7	284.3 ± 11	80.6 ± 8	32 ± 4.1	5.1 ± 0.4	2.8 ± 0.1	1.85 ± 0.09
	15 ppm	15.2 ± 1.1	291 ± 14	84.5 ± 10	52.7 ± 7*	5.2 ± 0.3	2.9 ± 0.1	1.80 ± 0.06
	32 ppm	14.9 ± 0.9	311 ± 12	86.1 ± 9	56 ± 5.8*	5.1 ± 0.5	3.0 ± 0.2	1.82 ± 0.08
1 month	Control	15.1 ± 1.4	301 ± 8.9	82.8 ± 9	33 ± 3.5	4.7 ± 0.4	3.1 ± 0.1	1.78 ± 0.04
	15 ppm	15.5 ± 0.6	353 ± 10*	89.6 ± 11	61.6 ± 5.7*	4.9 ± 0.3	3.2 ± 0.4	1.70 ± 0.07
	32 ppm	15.5 ± 1.1	388 ± 13*	106.4 ± 8*	79 ± 6.8*	4.8 ± 0.4	3.0 ± 0.2	1.63 ± 0.09
2 months	Control	16.1 ± 1.4	314 ± 14	83 ± 9	33.7 ± 3.5	5.1 ± 0.1	3.14 ± 0.3	1.72 ± 0.03
	15 ppm	21.3 ± 2.5*	368 ± 8*	108.4 ± 13*	77.4 ± 2.7*	4.8 ± 0.8	3.5 ± 0.5*	1.26 ± 0.05*
	32 ppm	23 ± 1.8*	372 ± 15*	120.7 ± 11*	99.8 ± 6.8*	4.9 ± 0.3	3.6 ± 0.2*	1.21 ± 0.09*
3 months	Control	16.6 ± 0.8	329 ± 13	91.8 ± 11	35 ± 6	5.3 ± 0.5	2.9 ± 0.3	1.54 ± 0.08
	15 ppm	22.7 ± 1.5*	389 ± 12*	121.3 ± 13*	69 ± 8.9*	4.0 ± 0.4*	3.6 ± 0.2*	1.07 ± 0.06*
	32 ppm	24.2 ± 1.3*	410 ± 10*	167 ± 10*	86.1 ± 4.6*	3.7 ± 0.5*	3.8 ± 0.1*	0.96 ± 0.07*
4 months	Control	17.1 ± 1.2	319 ± 10	105.8 ± 12	39.4 ± 4.8	4.9 ± 0.3	3.3 ± 0.6	1.45 ± 0.09
	15 ppm	23.2 ± 1.8*	428 ± 12*	143 ± 15*	56.9 ± 5.6*	3.8 ± 0.4*	3.9 ± 0.5*	0.96 ± 0.06*
	32 ppm	25.1 ± 1.4*	444 ± 13*	195.2 ± 14*	67.3 ± 7*	3.3 ± 0.5*	4.5 ± 0.4*	0.80 ± 0.05*
5 months	Control	17.2 ± 1.4	322 ± 8.5	98.3 ± 10	41 ± 4.5	4.8 ± 0.6	3.4 ± 0.3	1.42 ± 0.06
	15 ppm	24.6 ± 1.3*	451 ± 11*	184.6 ± 13*	44.7 ± 5.2	2.9 ± 0.4*	4.3 ± 0.2*	0.93 ± 0.04*
	32 ppm	31.4 ± 1.1*	531 ± 17*	233.1 ± 14	50.3 ± 6.1	2.6 ± 0.3*	4.8 ± 0.4*	0.71 ± 0.05*

The results shown are the average of 5 different experiments ± SEM. (*) indicates a significant difference with respect to control groups by a one-way ANOVA followed by a Bonferroni test

### Effect of Cd exposure on epididymal adipose infiltration of pro- and anti-inflammatory macrophages

**Fig. 1:**
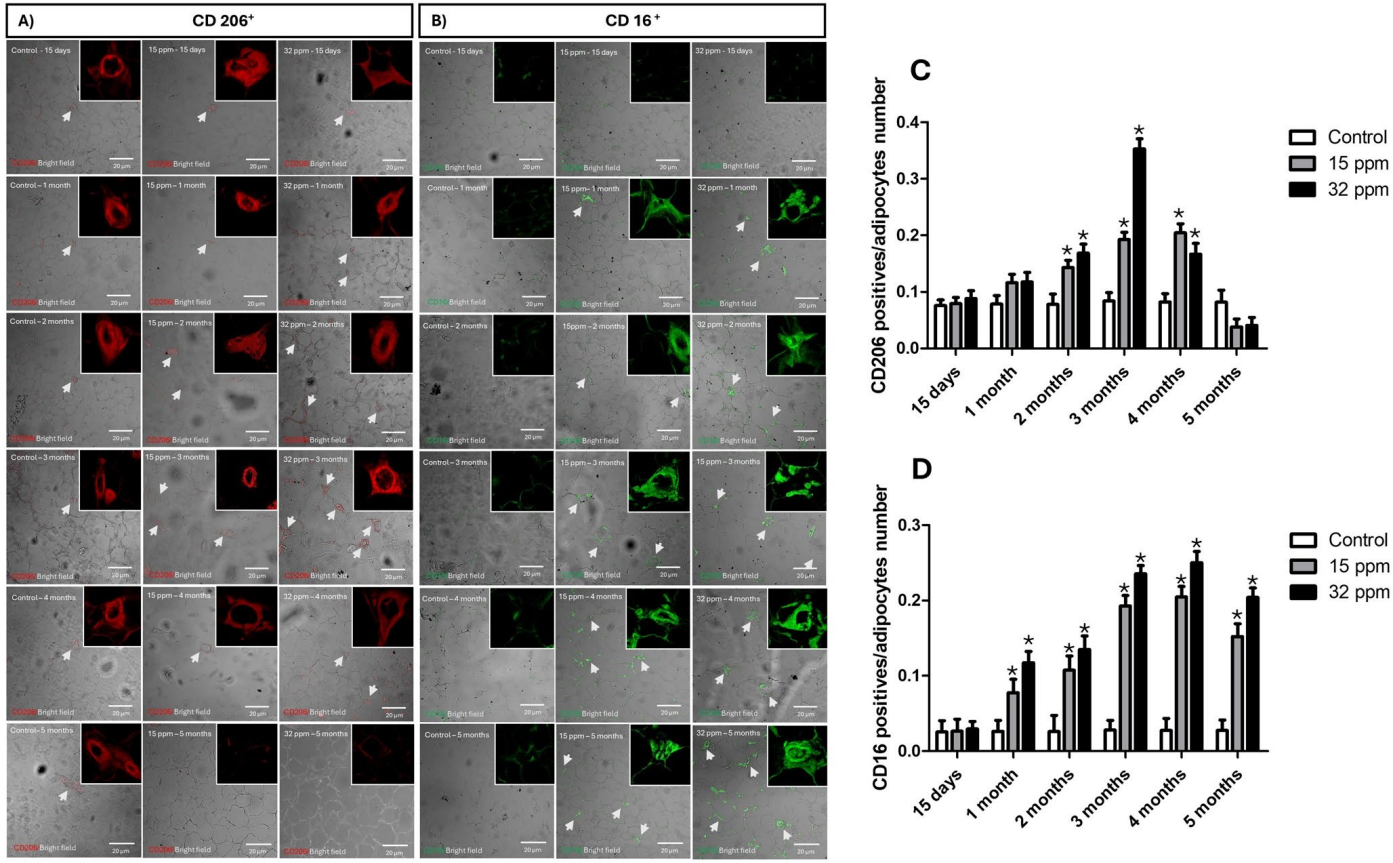
Effect of cadmium exposure on epididymal adipose expression of Pro and anti-inflammatory macrophages. **A** Representative images of CD206-positive. **B** CD206 positive cells by number of adipocytes. **C** Representative images of CD16-positive cells. **D** CD16-positive cells by number of adipocytes. The results shown are the average of 5 different experiments ± SEM. (*) indicates a significant difference regarding control groups (*p* ≤ 0.05) by a Kruskal–Wallis test

The expression of anti-inflammatory (CD206⁺) and pro-inflammatory macrophages (CD16⁺) was evaluated in epididymal adipose tissue (Fig. 1A–D). The infiltration of inflammatory macrophages increased from the first month of Cd exposure in the groups, reaching 646.9% in the 15 ppm group and 812.5% in the 32 ppm group. However, CD206⁺ macrophages observed a maximal increase at 3 months of analysis, in both groups by 148.0% and 230.8%, respectively. In the consecutive time-cohort analysis, anti-inflammatory macrophages decreased even below the control group at 5 months.

### Cadmium exposure induces overexpression of NF-κB in the epididymal adipose

**Fig. 2:**
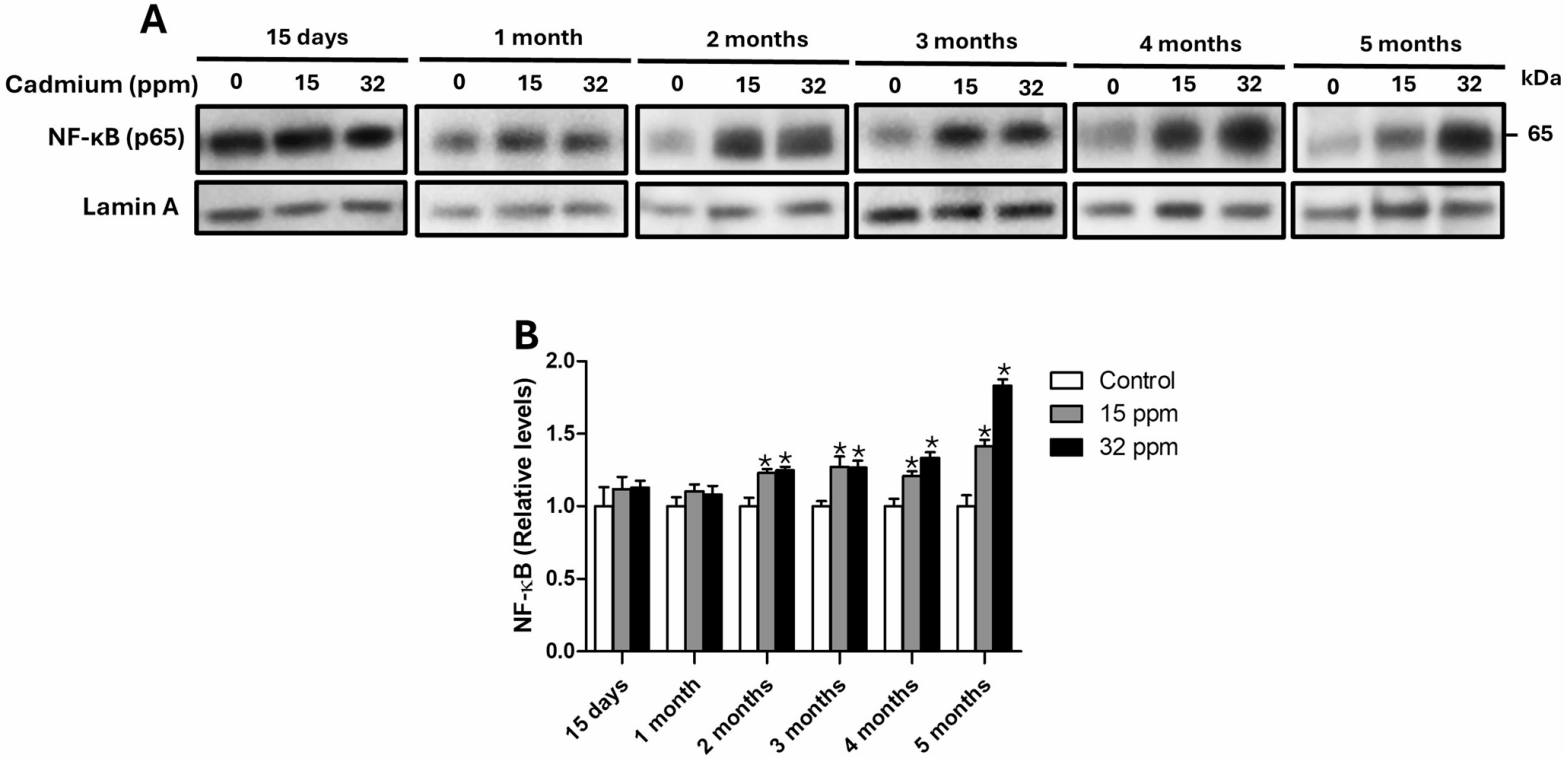
Effect of cadmium exposure on epididymal adipose expression of NF-κB. **A** Western transfer analysis of the expression of the protein NF-κB in nuclear extracts of the epididymal adipose tissue. **B** Densitometric analysis of NF-κB expression (normalized values). The results shown are the average of 5 different experiments ± SEM. (*) indicates a significant difference regarding control groups (*p* ≤ 0.05) by a one-way ANOVA followed by a Bonferroni post-test

The expression of NF-κB in epididymal adipose tissue increased over time, but was not Cd dose-dependent until five months of exposure (Fig. 2A and B). In the group exposed to 15 ppm, a significant nuclear increase of 10.4% was observed starting at the second month and sustained over time: 22.8% (2 months), 27.0% (3 months), 20.7% (4 months), and 41.4% (5 months). Similarly, in the 32 ppm group, NF-κB expression increased progressively: 8.3% (2 months), 24.7% (3 months), 26.5% (4 months), 33.3% (5 months), reaching a peak of 83.1% at the fifth month of exposure.

### Effect of cadmium exposure on epididymal adipose inflammatory and anti-inflammatory cytokines

**Fig. 3:**
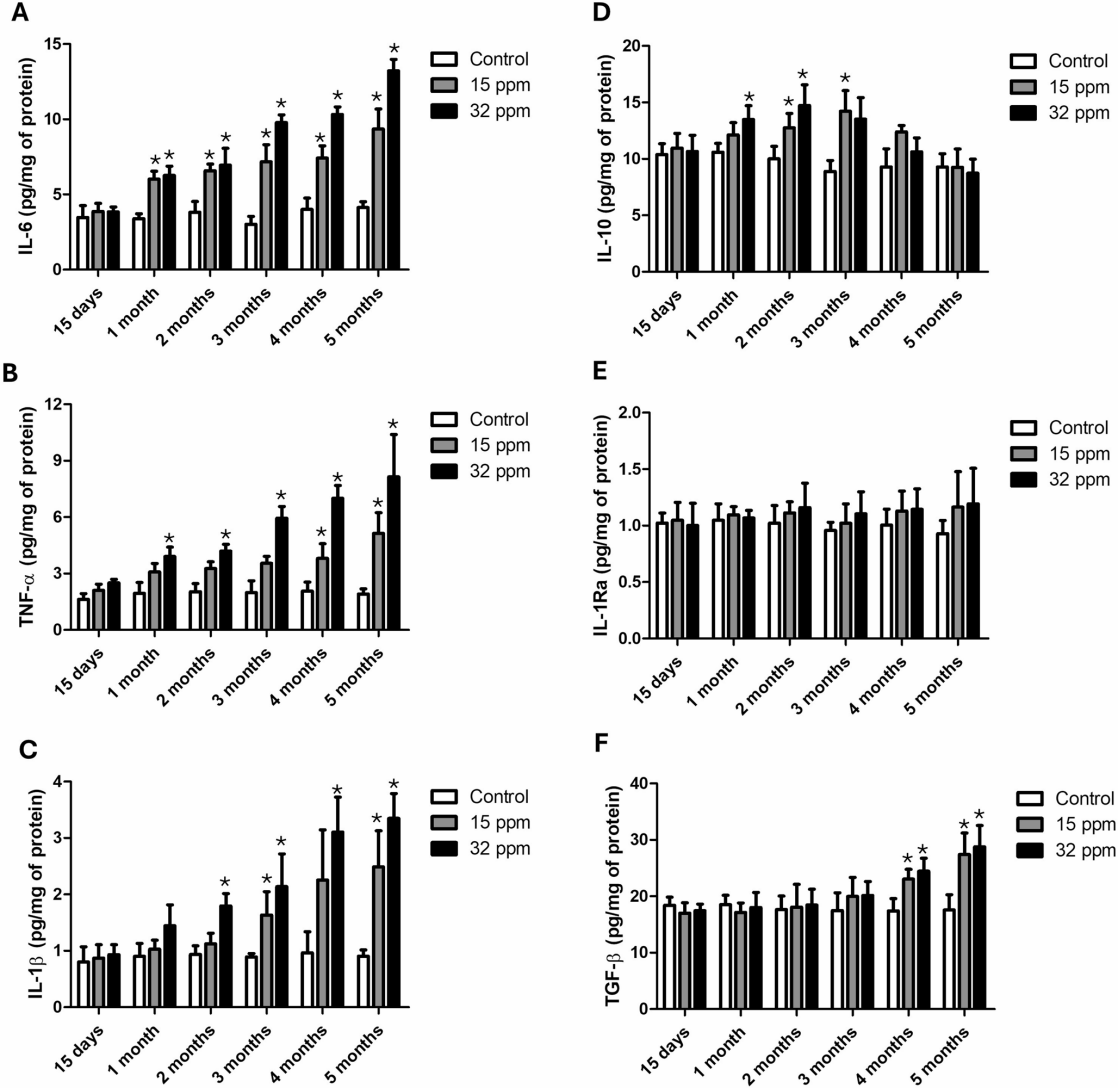
Effect of cadmium exposure on epididymal adipose inflammation and anti-inflammation. **A** IL-6; **B** TNF-α; **C** IL-1β; **D** IL-10; **E** IL-1Ra; **F** TGF-β. The results shown are the average of five different experiments ± SEM. (*) indicates a significant difference regarding control groups (*p* ≤ 0.05) by a one-way ANOVA followed by a Bonferroni post-test

The IL-6 concentration in epididymal adipose tissue increased significantly and progressively from the first month of exposure (Fig. 3A). In the 15 ppm group, IL-6 levels rose by 73.9% to 126.7%, and in the 32 ppm group by 84.5% to 220.2%. TNF-α exhibited the same dynamic, but it was more pronounced in the 32 ppm group (Fig. 3B). IL-1β increased from the third month onward by 83.7% to 175.3% in the 15 ppm group and from the second month in the 32 ppm group by 91.9% to 270.3% (Fig. 3C). In contrast, the anti-inflammatory cytokine IL-10 increased in the 15 ppm group starting in the second month (27.5%), peaking at the third month (59.3%), followed by a gradual decline in the fourth and fifth months. The 32 ppm group showed a similar pattern, with increases from the first month (27.3%), reaching 47.2% (2 months) and 51.9% (3 months), followed by a decline from the fourth month onward (Fig. 3D). IL-1Ra did not differ between experimental and control groups (Fig. 3E). Finally, TGF-β levels increased only in the last time cohort. In both Cd exposure groups, the levels increased by 32.3% and 55.7% (15 ppm), and 40.3% and 63.5% (32 ppm) at the fourth and fifth months (Fig. 3F).

### Cadmium exposure induces fibrosis in the epididymal adipose tissue

**Fig. 4:**
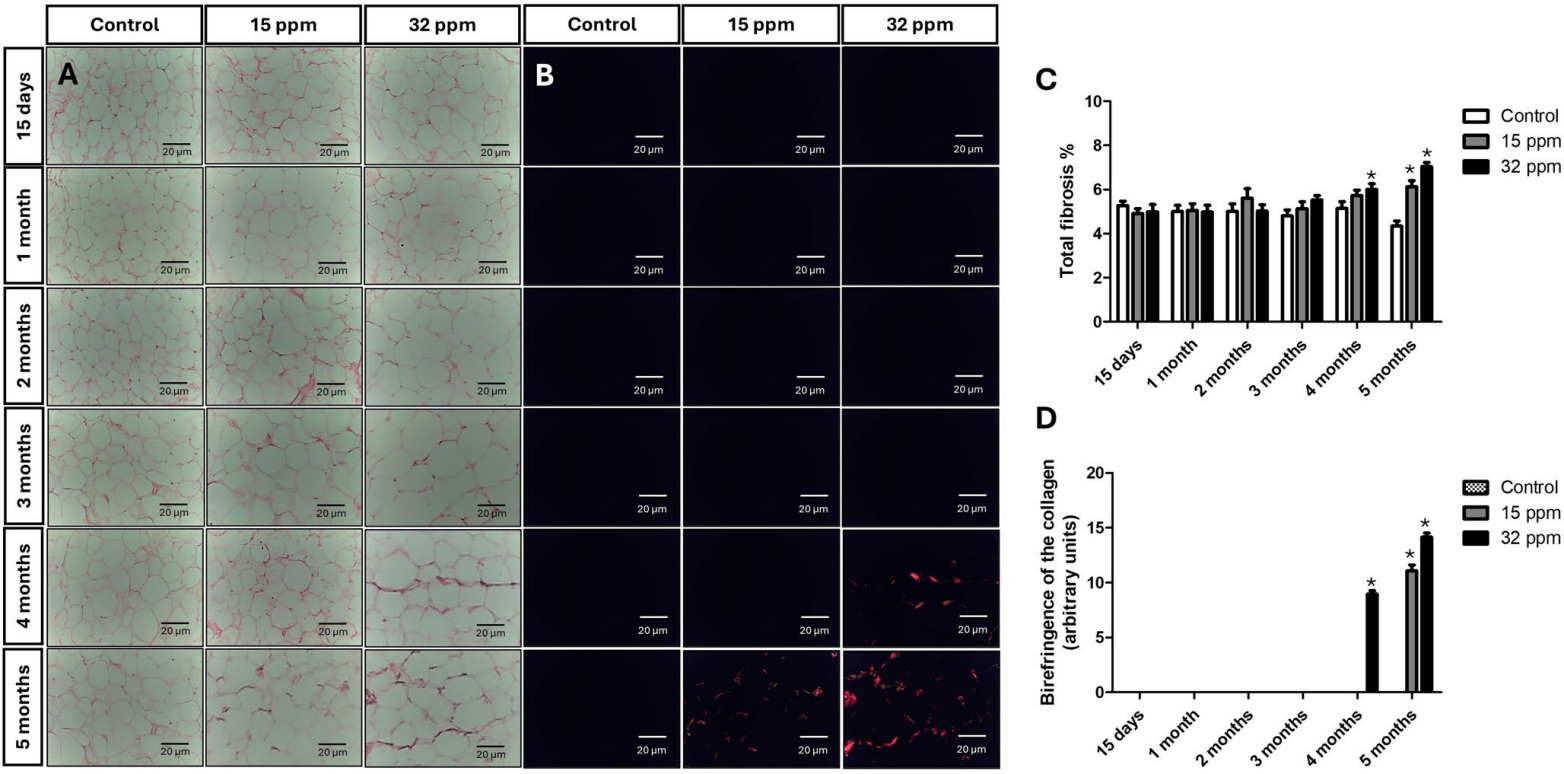
Effect of cadmium exposure on collagen deposition in epididymal adipose tissue using Picrosirius red staining. **A** Collagen (red fibers) in epididymal adipose tissue. **B** Collagen by polarized light to detect birefringence. **C** Percentage of fibrosis (red fibers) per field. **D** Quantification of the Birefringence for collagen. The results shown are the average of 5 different experiments ± SEM. (*) indicates a significant difference regarding control groups (*p* ≤ 0.05) by a Kruskal–Wallis test

Fibrosis in epididymal adipose tissue was evaluated by picrosirius red staining (Fig. 4A). A significant fibrosis deposit of 20.5% was observed only for 4 months in the 32 ppm group. At 5 months, epididymal adipose tissue fibrosis was more evident in both Cd exposure groups, by 41.3% in the 15 ppm group and by 126.0% in the 32 ppm group (Fig. 4B). Additionally, collagen birefringence under polarized light—indicative of organized collagen fiber deposition—was detected only at 4 months in the 32 ppm group, and at 5 months in both Cd exposure groups (Fig. 4C).

### Effects of Cd exposure on leptin and adiponectin expression in epididymal adipose tissue

**Fig. 5:**
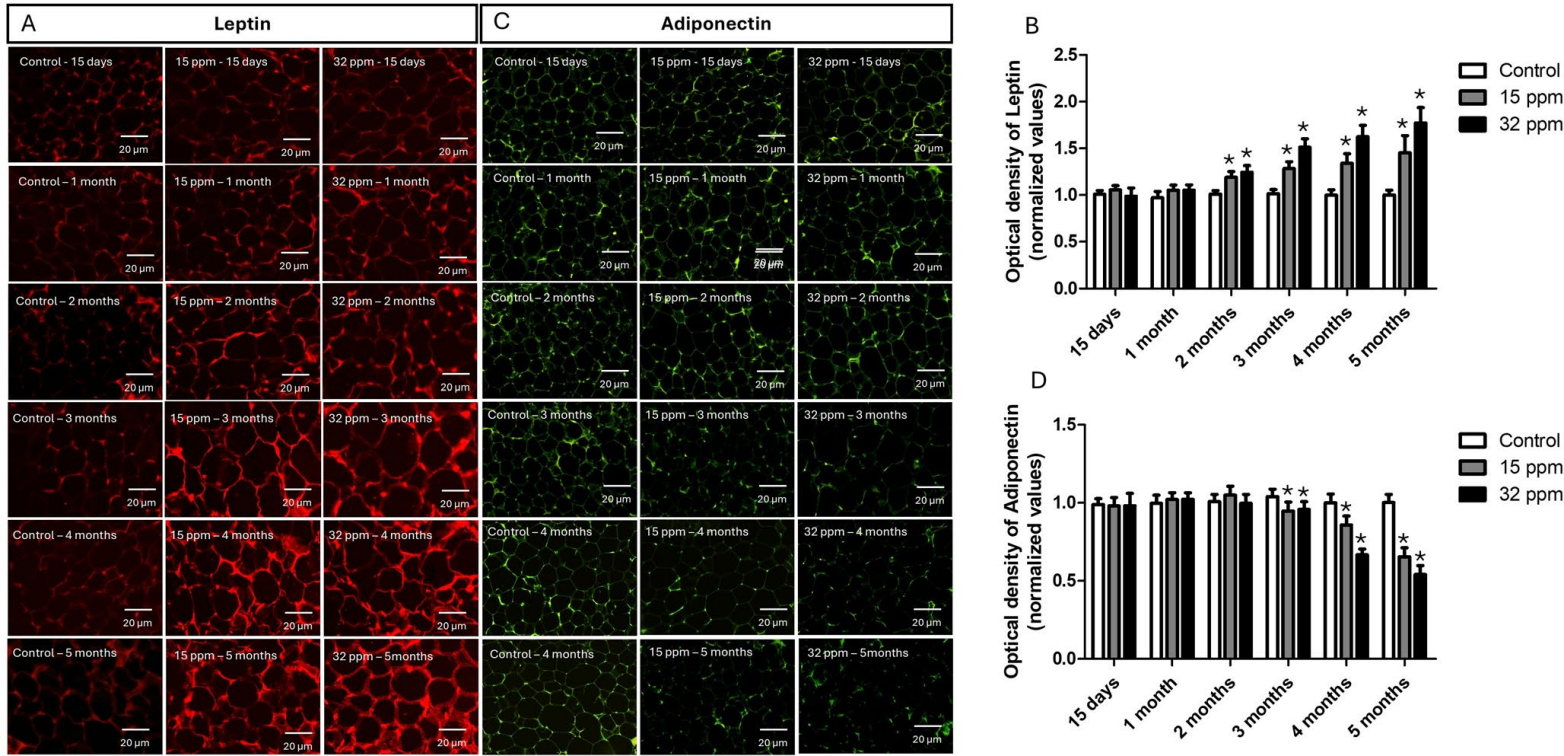
Effects of cadmium exposure on leptin and adiponectin expression in epididymal adipose tissue. **A** Leptin immunofluorescence expression. **B** Densitometric analysis of Leptin expression (normalized values). **C** Adiponectin immunofluorescence expression. **D** Densitometric analysis of adiponectin expression (normalized values). The results shown are the average of 5 different experiments ± SEM. (*) indicates a significant difference regarding control groups (*p* ≤ 0.05) by a Kruskal–Wallis test

Leptin expression in epididymal adipose tissue increased progressively with Cd exposure, time- and dose-dependent (Fig. 5A). An increase immunoreactivity was observed in both Cd exposure groups from the second month by 18.2% and 24.3%, reaching 45.7% and 77.8% (Fig. 5B). In contrast, adiponectin expression declined over time (Fig. 5C), with statistically significant reductions observed from 3 months in both Cd exposure groups 6.1% and 7.8% to reach 35.4% and 46.6% in the fifth month (Fig. 5D), which was more drastic in the 32 ppm group. These results strongly suggest a WAT dysfunction.

### Pearson’s correlation coefficients between the adiponectin/leptin ratio versus pro-inflammatory and anti-inflammatory markers after Cd exposure

**Fig. 6:**
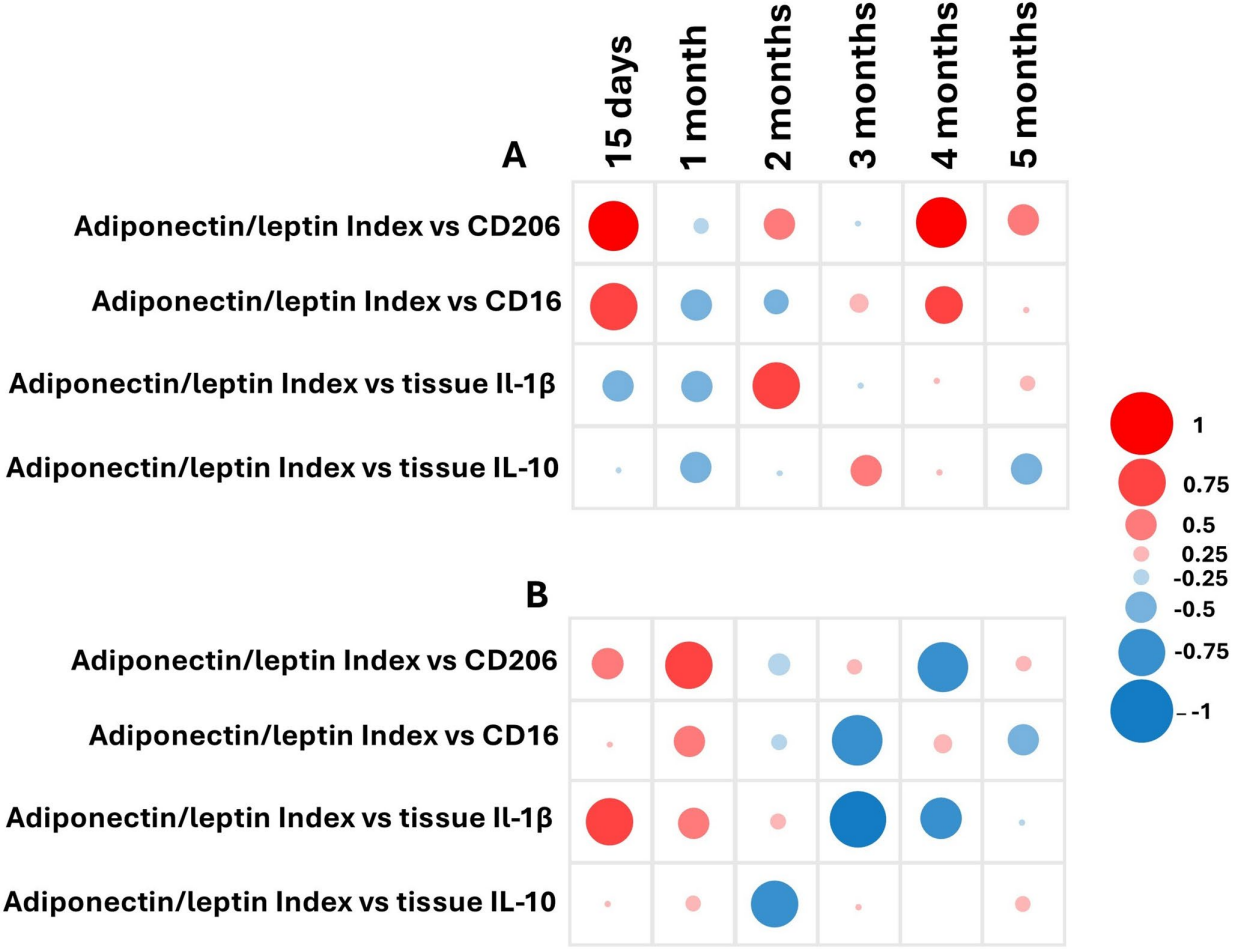
Heat map showing Pearson’s correlation coefficients between the Adiponectin/leptin ratio versus pro-inflammatory and anti-inflammatory markers after cadmium exposure. Significant correlations are colored either in red (positive) or blue (negative) hues. Numerical values are shown in Supplemental Table S1

Pearson’s correlation coefficients, which evaluate the relationship between the adiponectin/leptin ratio and pro-inflammatory (IL-1β and CD16) and anti-inflammatory (IL-10 and CD206) markers following Cd exposure. The data is stratified by dose (15 ppm and 32 ppm) and exposure duration (15 days, 1, 2, 3, 4, and 5 months). In the 15 ppm group, a strong positive correlation was observed between the adiponectin/leptin ratio and CD206 at 15 days (0.814) and 4 months (0.821). Similarly, strong positive correlations were found with CD16 at 15 days (0.672) and 4 months (0.629). A strong positive correlation with IL-1β (0.751) was also noted at 2 months. Meanwhile, the adiponectin/leptin ratio showed a negative correlation with IL-1β at 15 days and 1 month (− 0.445 and − 0.456), with IL-10 at 15 days (− 0.104), 1 month (− 0.557), and 5 months (− 0.635) (Fig. 6).

Cadmium exposure to 32 ppm showed a positive correlation with IL-1β at 15 days and 1 month (0.740 and 0.429). The ratio also showed a positive correlation with CD206 at 15 days (0.460) and 1 month (0.724). However, the analysis showed a negative correlation with IL-1β at 3 and 4 months (− 0.899 and − 0.662). Negative correlations were found with IL-10 at 2 and 5 months (− 0.736 and − 0.304, respectively). The ratio was also negatively correlated with CD16 at 3 and 5 months (− 0.860 and − 0.547).

## Discussion

In this work, we investigated the relationship between the immunological, structural, and functional epididymal WAT alterations of male Wistar rats induced by chronic exposure to environmental doses of Cd over time. Previously, we reported that Cd at a minimal risk dose is accumulated in epididymal WAT, leading to adipocyte hypertrophy, selective insulin resistance, high triglyceride storage, and the release of free fatty acids, as well as poor glycogen levels and impaired dynamic synthesis/release of adipokines. Also, we demonstrated that adipose insulin resistance increased simultaneously with lipolysis and glucose intolerance despite high glucose transporter 4 expression (Sarmiento-Ortega et al. 2025a ). We have provided strong evidence that chronic Cd exposure at environmental levels has toxic effects, which are independent of oxidative stress and contribute to the development of metabolic disorders (Sarmiento-Ortega et al. 2022, 2023; Moroni-González et al. 2023, 2024). However, it remains unclear whether this is due to adiposopathy (adipocyte dysfunction) and inflammatory/immunologic changes in adipocytes, as seen in metabolic diseases resulting from hypercaloric diet consumption or sedentary lifestyle.

In this sense, adipose tissue is increasingly recognized as a key immunometabolic organ, whose function extends far beyond energy storage. Chronic inflammation within adipose tissue, characterized by cytokine overproduction and adipokine imbalance, such as in adiposopathy, plays a key role in the development of insulin resistance, type 2 diabetes, and other metabolic disorders (Kawai et al. 2021; Kolb 2022; Tilg et al. 2025). WAT inflammation has been extensively studied in the context of obesity and overnutrition; however, its modulation by environmental toxicants such as Cd remains unexplored. The present study demonstrates that chronic Cd exposure induces a progressive and sustained systemic pro-inflammatory response (Table 1). This was evidenced by a serum increase of IL-6, IL-1β, and TNF-α levels, accompanied by dynamic alterations in anti-inflammatory cytokines such as IL-10 and TGF-β. The cytokine pattern could be the cause or consequence of inflammation in other tissues. It has been described how Cd can alter both innate and adaptive immunity, promoting the production of pro-inflammatory cytokines (IL-1β, IL-6, TNF-α), the activation of signaling pathways such as, the mitogen-activated protein kinases (MAPKs) and phosphoinositol 3-kinase (PI3K)/ protein kinase B (Akt), and the increase of reactive oxygen species (ROS), which contributes to tissue damage and organ dysfunction which appear to be dose, time, and tissue dependent, with some conflicting evidence suggesting immunosuppressive effects (Arroyo et al. 2013; Das and Al-Naemi 2019; Hossein-Khannazer et al. 2020). Although its inflammatory role in organs such as the liver, lungs, intestine, and kidneys has been documented, its inflammatory effect on adipose tissue remains poorly studied.

Adipose tissue is composed of a variety of cells, including adipocytes, endothelial cells, fibroblasts, and immune cells. Adipose tissue macrophages (ATMs) are essential for maintaining homeostasis by removing dead adipocytes and taking up lipids during lipolysis (Chavakis et al. 2023; Kado et al. 2024). Our results showed that Cd exposure increases circulating pro-inflammatory cytokines, accompanied by dynamic changes in anti-inflammatory cytokines. These changes were paralleled by a marked infiltration of pro-inflammatory CD16⁺ macrophages and a reduction in CD206⁺ macrophages over time, which is another hallmark of adiposopathy (Fig. 1). Attia et al. found that after exposure to CdCl_2_ at a dose of 15 mg of Cd/kg of body weight for 10 weeks, the expression of monocyte chemotactic protein 1 (MCP-1) mRNA decreased in retroperitoneal and subcutaneous adipose tissue, while an upregulation of the abdominal adipose tissue was observed. In addition, they also observed trends of positive or negative regulation in the expression of TNF-α, IL-6, and IL-10 according to the location of adipose tissue, suggesting a differential inflammatory response induced by Cd according to the location of adipose tissue (Attia et al. 2022a). Despite limited information, we believe that the change in macrophage phenotype and inflammatory profile is due to the accumulation of metal in the tissue, even at doses of minimal risk, which modulates the redox balance, leads to insulin resistance, and alters the signaling pathway of this hormone (Sarmiento-Ortega et al. 2022, 2025b).

The pro-inflammatory cytokines activate the NF-κB signaling pathway, which promotes the transcription of proteins and mediators involved in inflammatory processes, thus intensifying inflammation in adipose tissue (Griffin 2022). The activation of the NF-κB signaling pathway plays a central role in modulating macrophage polarization within adipose tissue (Hill et al. 2015). Our results showed that the expression of NF-κB in adipose tissue is dose and time-dependent on the concentration of Cd exposed (Fig. 2) and may be a cause and/or consequence of the observed macrophage polarization. A study in hens after exposure to 150 mg/k of CdCl_2_ for 60 days showed an increase in the expression of the NF-κB transcript in adipose tissue, accompanied by a pro-oxidant, pro-inflammatory environment regulated by heat shock proteins (Sun et al. 2023). Together, these findings suggest a disruption by Cd exposure of adipose immune homeostasis and a shift toward an unresolved inflammatory state, as can be evidenced in our results of the concentration of these cytokines directly in adipose tissue (Fig. 3). The data presented reveal a progressive and dose-dependent increase in pro-inflammatory cytokines (IL-6, TNF-α, TGF-β, and IL-1β) in adipose tissue following Cd exposure, which correlates with the increased presence of CD16⁺ (M1) macrophages and enhanced NF-κB activation. Although IL-10 showed transient elevation in early stages, its decline in later time points indicates an exhaustion of anti-inflammatory responses, consistent with the observed reduction in CD206⁺ (M2-like) macrophages. This phenotypic switch not only amplifies local inflammation but also contributes to extracellular matrix remodeling. In particular, the sustained presence of M1 macrophages and elevated inflammatory signaling, mainly by a late increase in TGF-β.

It is widely recognized that TGF-β promotes collagen deposition and fibrotic responses that generate tissue remodeling and extracellular matrix accumulation (ECM). Consistent with this, our findings revealed increased collagen content and birefringence in late stages of cadmium exposure (Fig. 4), suggesting that chronic inflammation mediated by NF-κB, as well as activation and macrophage dysfunction, is closely associated with the development of adipose tissue fibrosis, which is a distinctive feature of adiposopathy. Collagen in adipose tissue fulfills dual functions: at physiological levels, it supports the architecture and metabolic functioning of the tissue, but its excessive accumulation, as occurs in fibrosis, compromises its flexibility and lipid storage capacity, favoring metabolic dysfunction (Datta et al. 2018). Fibrosis in adipose tissue represents a key feature of its dysfunction in contexts such as obesity, where ECM accumulates excessively, limiting tissue expansion and favoring chronic inflammation and insulin resistance. Macrophages play a dual role, contributing to both collagen production and purification, with the activation of toll-like receptor 4 (TLR4), TGF-β/Smad, NF-κB, and MAPKs being the key mechanisms in fibrogenesis. In addition, fibrosis imposes physical restrictions that modify adipocyte function through mechanosensitive pathways, altering adipokine secretion and decreasing insulin sensitivity (Datta et al. 2018; Marcelin et al. 2022; Gliniak et al. 2023; Eisinger et al. 2024; Dahdah et al. 2024).

Cadmium exposure has been associated with the development of fibrosis in different organs, including the kidney, liver, and lungs. In animal models, Cd has been shown to activate inflammatory and profibrotic pathways in kidneys such as NF-κB, MAPK, and TGF-β/Smad, promoting fibroblast proliferation, collagen synthesis, and ECM accumulation (Joardar et al. 2019). In the kidney and liver, these effects are aggravated under conditions of oxidative stress or Nrf2 deficiency (Chen et al. 2023; Xu et al. 2025). Although less studied, Cd has also been reported to induce fibrosis in adipose tissue. A study with subacute exposure to high concentrations of Cd (100 ppm for 30 days) in rats demonstrated a significant increase in fibrosis in adipose tissue, accompanied by inflammation and apoptosis (da Costa et al. 2024). Our findings become relevant by demonstrating that fibrosis in adipose tissue can develop even with doses of minimal risk, although in a progressive way, which highlights the sensitivity of adipose tissue to prolonged exposures to environmentally relevant and subclinical doses. Furthermore, the final puzzle piece to evidence adiposopathy develop is a reduction of adiponectin levels, coupled with increased leptin expression both in serum and in adipose tissue (Fig. 5), supporting the notion of adipose tissue dysfunction and a potentially insulin-resistant phenotype, mediated by inflammation and by observed fibrosis, which reinforce the hypothesis that Cd disrupts immunometabolic interactions in a time- and dose-dependent manner, but reinforces the importance of considering the cumulative Cd effects in tissue remodeling processes.

Finally, we analyzed our most important results in a correlation map to identify dynamic patterns and relevant biochemical/clinical associations in our study. The results show that the balance between adiponectin and leptin, represented by the A/L index, is closely linked to the immune status of adipose tissue and that this relationship is profoundly altered by chronic Cd exposure. Under normal conditions, a high A/L index is usually associated with an anti-inflammatory profile, while a low index is linked to inflammation and adipocyte dysfunction (Frühbeck et al. 2018). The data show that the relationship between the A/L ratio and inflammatory biomarkers is dynamic and time-dependent, with differences between Cd exposure to 15 ppm and 32 ppm. While at 15 ppm, a mostly positive correlation with CD206 was observed, at 32 ppm, correlations with IL-1β suggest a progressive inflammatory response to a greater concentration of Cd exposure. The correlations observed reveal that under Cd exposure (especially at 32 ppm), the A/L index loses its protective relationship and inverts or weakens as the exposure time progresses. For example, the transition from positive to negative correlations with IL-1β and CD16 suggests that, despite an eventual early compensatory response, the tissue environment ends up favoring the activation of proinflammatory macrophages and the production of cytokines such as IL-1β, indicative of chronic inflammation. Loss of correlation with IL-10 and CD206 in advanced stages suggests depletion or suppression of the anti-inflammatory axis, likely due to persistence of the toxic stimulus and failure to resolve inflammation. This aligns with the concept of “dysfunctional immunometabolism” of adipose tissue, where hormonal signaling and innate immunity are decoupled, losing their regulatory synchrony (Kumari et al. 2018; Trim et al. 2018; Basso et al. 2025).

In summary, we demonstrate that chronic Cd exposure to environmental doses progressively induces inflammation in adipose tissue, evidenced by increased pro-inflammatory cytokines (IL-6, TNF-α, IL-1β), activation of the NF-κB pathway, infiltration of M1 macrophages (CD16⁺), and reduction of M2 macrophages (CD206⁺). These immunological changes were accompanied by endocrine dysfunction of the tissue, reflected in the imbalance of the leptin/adiponectin axis and the decrease in the A/L index. In addition, a late increase in TGF-β and development of fibrosis were observed in chronicity. Dynamic correlations between the A/L ratio and immune biomarkers suggest a progressive loss of tissue immunometabolic control. Hallmarks of adiposopathy were evidenced for the first time, induced by Cd exposure. In conclusion, Cd exposure at low concentrations deteriorates the immunometabolic homeostasis of adipose tissue and promotes an inflammatory and profibrotic environment, contributing to adiposopathy. These findings reinforce the need to consider adipose tissue as a sensitive target for environmental pollutants in the development of metabolic diseases.

## Supplementary Information

Below is the link to the electronic supplementary material.

Supplementary Material 1

## References

[CR1] Arroyo VS, Flores KM, Ortiz LB et al (2013) Liver and cadmium toxicity. J Drug Metab Toxicol. 10.4172/2157-7609.S5-001

[CR2] Attia SM, Das SC, Varadharajan K, Al-Naemi HA (2022a) White adipose tissue as a target for cadmium toxicity. Front Pharmacol. 10.3389/fphar.2022.1010817 36278208 10.3389/fphar.2022.1010817 PMC9582776

[CR3] Attia SM, Varadharajan K, Shanmugakonar M et al (2022b) Cadmium: an emerging role in adipose tissue dysfunction. Expo Health 14:171–183. 10.1007/s12403-021-00427-3

[CR4] Basso PJ, Schcolnik-Cabrera A, Zhu M et al (2025) Weight loss-associated remodeling of adipose tissue immunometabolism. Obes Rev. 10.1111/obr.13975 40589031 10.1111/obr.13975 PMC12620110

[CR5] Blüher M (2024) Understanding adipose tissue dysfunction. J Obes Metab Syndr 33:275–288. 10.7570/jomes24013 39734091 10.7570/jomes24013 PMC11704217

[CR6] Chavakis T, Alexaki VI, Ferrante AW (2023) Macrophage function in adipose tissue homeostasis and metabolic inflammation. Nat Immunol 24:757–766. 10.1038/s41590-023-01479-0 37012544 10.1038/s41590-023-01479-0

[CR7] Chen C, Zhou Z, Yu S et al (2023) Nrf2 protects against renal fibrosis induced by chronic cadmium exposure in mice. Food Chem Toxicol 178:113875. 10.1016/j.fct.2023.113875 37286028 10.1016/j.fct.2023.113875

[CR8] Chung SM, Chang MC (2025) Cadmium exposure and thyroid hormone disruption: a systematic review and meta-analysis. Rev Environ Health 40:37–46. 10.1515/reveh-2023-0122 38142367 10.1515/reveh-2023-0122

[CR9] Coelho PGB, de Souza MV, Conceição LG et al (2018) Evaluation of dermal collagen stained with picrosirius red and examined under polarized light microscopy. An Bras Dermatol 93:415–418. 10.1590/abd1806-4841.20187544 29924246 10.1590/abd1806-4841.20187544 PMC6001092

[CR10] da Costa CS, de Oliveira TF, Dos Santos FCF et al (2024) Subacute cadmium exposure changes different metabolic functions, leading to type 1 and 2 diabetes mellitus features in female rats. Environ Toxicol 39:4278–4297. 10.1002/tox.24306 38712533 10.1002/tox.24306

[CR11] Dagdag O, Quadri TW, Haldhar R et al (2023) An overview of heavy metal pollution and control. pp 3–24

[CR12] Dahdah N, Tercero-Alcázar C, Malagón MM et al (2024) Interrelation of adipose tissue macrophages and fibrosis in obesity. Biochem Pharmacol 225:116324. 10.1016/j.bcp.2024.116324 38815633 10.1016/j.bcp.2024.116324

[CR13] Das SC, Al-Naemi HA (2019) Cadmium toxicity: oxidative stress, inflammation and tissue injury. Occup Dis Environ Med 07:144–163. 10.4236/odem.2019.74012

[CR14] Datta R, Podolsky MJ, Atabai K (2018) Fat fibrosis: friend or foe? JCI Insight. 10.1172/jci.insight.122289 30282827 10.1172/jci.insight.122289 PMC6237440

[CR15] Edwards J, Ackerman C (2016) A review of Diabetes mellitus and exposure to the environmental toxicant Cadmium with an emphasis on likely mechanisms of action. Curr Diabetes Rev 12:252–258 26264451 10.2174/1573399811666150812142922 PMC5002940

[CR16] Edwards JR, Prozialeck WC (2009) Cadmium, diabetes and chronic kidney disease. Toxicol Appl Pharmacol 238:289–293. 10.1016/j.taap.2009.03.007 19327375 10.1016/j.taap.2009.03.007 PMC2709710

[CR17] Eisinger K, Girke P, Buechler C, Krautbauer S (2024) Adipose tissue depot specific expression and regulation of fibrosis-related genes and proteins in experimental obesity. Mamm Genome 35:13–30. 10.1007/s00335-023-10022-3 37884762 10.1007/s00335-023-10022-3 PMC10884164

[CR18] Frühbeck G, Catalán V, Rodríguez A, Gómez-Ambrosi J (2018) Adiponectin-leptin ratio: a promising index to estimate adipose tissue dysfunction. Relation with obesity-associated cardiometabolic risk. Adipocyte 7:57–62. 10.1080/21623945.2017.1402151 29205099 10.1080/21623945.2017.1402151 PMC5915018

[CR19] Gliniak CM, Pedersen L, Scherer PE (2023) Adipose tissue fibrosis: the unwanted houseguest invited by obesity. J Endocrinol. 10.1530/JOE-23-0180 37855264 10.1530/JOE-23-0180 PMC11648981

[CR20] Griffin MJ (2022) On the immunometabolic role of NF-κB in adipocytes. Immunometabolism 4:10.20900/immunometab20220003 10.20900/immunometab20220003 PMC8893669 35251704

[CR21] Hill AA, Anderson-Baucum EK, Kennedy AJ et al (2015) Activation of NF-κB drives the enhanced survival of adipose tissue macrophages in an obesogenic environment. Mol Metab 4:665–677. 10.1016/j.molmet.2015.07.005 26779432 10.1016/j.molmet.2015.07.005 PMC4588436

[CR22] Hossein-Khannazer N, Azizi G, Eslami S et al (2020) The effects of cadmium exposure in the induction of inflammation. Immunopharmacol Immunotoxicol 42:1–8 31793820 10.1080/08923973.2019.1697284

[CR23] Huang Y, Hu Y, Chen H et al (2024) Anatomy and physiology of adipose tissue. pp 47–92

[CR24] Joardar S, Dewanjee S, Bhowmick S et al (2019) Rosmarinic acid attenuates Cadmium-induced nephrotoxicity via inhibition of oxidative stress, apoptosis, inflammation and fibrosis. Int J Mol Sci 20:2027. 10.3390/ijms20082027 31022990 10.3390/ijms20082027 PMC6514581

[CR25] Kado T, Nishimura A, Tobe K (2024) History and future perspectives of adipose tissue macrophage biology. Front Pharmacol. 10.3389/fphar.2024.1373182 38562458 10.3389/fphar.2024.1373182 PMC10982364

[CR26] Kawai T, Autieri MV, Scalia R (2021) Adipose tissue inflammation and metabolic dysfunction in obesity. Am J Physiol Cell Physiol 320:C375–C391. 10.1152/ajpcell.00379.2020 33356944 10.1152/ajpcell.00379.2020 PMC8294624

[CR27] Kolb H (2022) Obese visceral fat tissue inflammation: from protective to detrimental? BMC Med 20:494. 10.1186/s12916-022-02672-y 36575472 10.1186/s12916-022-02672-y PMC9795790

[CR28] Kubier A, Wilkin RT, Pichler T (2019) Cadmium in soils and groundwater: a review. Appl Geochem 108:104388. 10.1016/j.apgeochem.2019.104388 10.1016/j.apgeochem.2019.104388 PMC7147761 32280158

[CR29] Kumari M, Heeren J, Scheja L (2018) Regulation of immunometabolism in adipose tissue. Semin Immunopathol 40:189–202. 10.1007/s00281-017-0668-3 29209828 10.1007/s00281-017-0668-3

[CR30] Lopez-Yus M, Hörndler C, Borlan S et al (2024) Unraveling adipose tissue dysfunction: molecular mechanisms, novel biomarkers, and therapeutic targets for liver fat deposition. Cells 13:380. 10.3390/cells13050380 38474344 10.3390/cells13050380 PMC10931433

[CR31] Luevano J, Damodaran C (2014) A review of molecular events of cadmium-induced carcinogenesis. J Environ Pathol Toxicol Oncol 33:183–194 25272057 10.1615/jenvironpatholtoxicoloncol.2014011075 PMC4183964

[CR32] Marcelin G, Gautier EL, Clément K (2022) Adipose tissue fibrosis in obesity: etiology and challenges. Annu Rev Physiol 84:135–155. 10.1146/annurev-physiol-060721-092930 34752708 10.1146/annurev-physiol-060721-092930

[CR33] Moroni-González D, Sarmiento-Ortega VE, Diaz A et al (2023) Pancreas–liver–adipose axis: target of environmental cadmium exposure linked to metabolic diseases. Toxics 11:223. 10.3390/toxics11030223 36976988 10.3390/toxics11030223 PMC10059892

[CR34] Moroni-González D, Sarmiento-Ortega VE, Diaz A et al (2024) Pancreatic antioxidative defense and heat shock proteins prevent islet of Langerhans cell death after chronic oral exposure to cadmium LOAEL dose. Biol Trace Elem Res 202:3714–3730. 10.1007/s12011-023-03955-y 37955768 10.1007/s12011-023-03955-y

[CR35] Reyes-Farias M, Fos-Domenech J, Serra D et al (2021) White adipose tissue dysfunction in obesity and aging. Biochem Pharmacol 192:114723. 10.1016/j.bcp.2021.114723 34364887 10.1016/j.bcp.2021.114723

[CR36] Sarmiento-Ortega VE, Alcántara-Jara DI, Moroni-González D et al (2025a) Chronic cadmium exposure to minimal-risk doses causes dysfunction of epididymal adipose tissue and metabolic disorders. Toxicol Appl Pharmacol 495:117203. 10.1016/J.TAAP.2024.117203 39701214 10.1016/j.taap.2024.117203

[CR37] Sarmiento-Ortega VE, Moroni-González D, Diaz A et al (2023) ROS and ERK pathway mechanistic approach on hepatic insulin resistance after chronic oral exposure to cadmium NOAEL dose. Biol Trace Elem Res 201:3903–3918. 10.1007/s12011-022-03471-5 36348173 10.1007/s12011-022-03471-5

[CR38] Sarmiento-Ortega VE, Moroni-González D, Díaz A et al (2022) Oral subacute exposure to cadmium LOAEL dose induces insulin resistance and impairment of the hormonal and metabolic liver-adipose axis in Wistar rats. Biol Trace Elem Res 200:4370–4384. 10.1007/s12011-021-03027-z 34846673 10.1007/s12011-021-03027-z

[CR39] Sarmiento-Ortega VE, Treviño S, Flores-Hernández JÁ et al (2017) Changes on serum and hepatic lipidome after a chronic cadmium exposure in Wistar rats. Arch Biochem Biophys 635:52–59. 10.1016/j.abb.2017.10.003 29066246 10.1016/j.abb.2017.10.003

[CR40] Satarug S (2023) Is environmental cadmium exposure causally related to diabetes and obesity? Cells 13:83. 10.3390/cells13010083 38201287 10.3390/cells13010083 PMC10778334

[CR41] Satarug S, Garrett SH, Sens MA, Sens DA (2010) Cadmium, environmental exposure, and health outcomes. Environ Health Perspect 118:182–190. 10.1289/ehp.0901234 20123617 10.1289/ehp.0901234 PMC2831915

[CR42] Satarug S, Vesey DA, Gobe GC, Phelps KR (2023) Estimation of health risks associated with dietary cadmium exposure. Arch Toxicol 97:329–358. 10.1007/s00204-022-03432-w 36592197 10.1007/s00204-022-03432-w

[CR43] Sun J, Jiao Z, Zhu W et al (2023) Astilbin attenuates cadmium-induced adipose tissue damage by inhibiting NF-κB pathways and regulating the expression of HSPs in chicken. Biol Trace Elem Res 201:2512–2523. 10.1007/s12011-022-03327-y 35717552 10.1007/s12011-022-03327-y

[CR44] Thévenod F, Lee W-K (2013) Toxicology of cadmium and its damage to mammalian organs. pp 415–490 10.1007/978-94-007-5179-8_14 23430781

[CR45] Tilg H, Ianiro G, Gasbarrini A, Adolph TE (2025) Adipokines: masterminds of metabolic inflammation. Nat Rev Immunol 25:250–265. 10.1038/s41577-024-01103-8 39511425 10.1038/s41577-024-01103-8

[CR46] Tinkov AA, Filippini T, Ajsuvakova OP et al (2017) The role of cadmium in obesity and diabetes. Sci Total Environ 601:741–755. 10.1016/j.scitotenv.2017.05.224 28577409 10.1016/j.scitotenv.2017.05.224

[CR47] Trim W, Turner JE, Thompson D (2018) Parallels in immunometabolic adipose tissue dysfunction with ageing and obesity. Front Immunol. 10.3389/fimmu.2018.00169 29479350 10.3389/fimmu.2018.00169 PMC5811473

[CR48] Xu C, Li Z, Hao S et al (2025) Association of blood cadmium levels with all-cause and cause-specific mortality among adults with non-alcoholic fatty liver disease: a prospective cohort study. Front Public Health. 10.3389/fpubh.2025.1573760 40255375 10.3389/fpubh.2025.1573760 PMC12006042

[CR49] Yang Loureiro Z, Solivan-Rivera J, Corvera S (2022) Adipocyte heterogeneity underlying adipose tissue functions. Endocrinology. 10.1210/endocr/bqab138 34223880 10.1210/endocr/bqab138 PMC8660558

[CR50] Yuan Z, Luo T, Liu X et al (2019) Tracing anthropogenic cadmium emissions: from sources to pollution. Sci Total Environ 676:87–96. 10.1016/j.scitotenv.2019.04.250 31029903 10.1016/j.scitotenv.2019.04.250

[CR51] Zhu Y, Cheng P, Peng J et al (2024) Cadmium exposure causes transcriptomic dysregulation in adipose tissue and associated shifts in serum metabolites. Environ Int 185:108513. 10.1016/j.envint.2024.108513 38382403 10.1016/j.envint.2024.108513

