# Supplementary material for: Oral cadmium exposure to environmental doses induces visceral adiposopathy in Wistar rats
Source: Arch Toxicol. 2025 Nov 7;100(2):451–63. doi: 10.1007/s00204-025-04228-4 (PMC12886319; doi:10.1007/s00204-025-04228-4)
Supplement: Supplementary file 1 — Supplementary Material 1 [file 204_2025_4228_MOESM1_ESM.docx]

| 15 ppm | | | | | | | | |
| --- | --- | --- | --- | --- | --- | --- | --- | --- |
|  | | Il-1b | | Il10 | | Cd206 | | Cd16 |
| 15 days | | -0.445 | | -0.104 | | 0.814 | | 0.672 |
| 1 month | | -0.456 | | -0.557 | | -0.515 | | -0.234 |
| 2 months | | 0.751 | | -0.119 | | 0.567 | | -0.419 |
| 3 months | | -0.134 | | 0.466 | | -0.189 | | 0.307 |
| 4 months | | 0.160 | | 0.154 | | 0.821 | | 0.629 |
| 5 months | | 0.234 | | -0.635 | | 0.393 | | 0.108 |
| 32 ppm | | | | | | | | |
|  | Il-1b | | Il10 | | Cd206 | | Cd16 | |
| 15 days | 0.740 | | 0.114 | | 0.460 | | 0.051 | |
| 1 month | 0.429 | | 0.373 | | 0.724 | | 0.499 | |
| 2 months | 0.362 | | -0.736 | | -0.362 | | -0.277 | |
| 3 months | -0.899 | | 0.117 | | 0.223 | | -0.860 | |
| 4 months | -0.662 | | 0.006 | | -0.802 | | 0.306 | |
| 5 months | 0.159 | | -0.304 | | 0.229 | | -0.547 | |

S1. Numerical values of Pearson’s correlation coefficients between the Adiponectin/leptin ratio versus pro-inflammatory and anti-inflammatory markers after cadmium exposure.
